# Massive hemoptysis as the sentinel symptom: a case report of pulmonary nocardiosis in an immunocompetent patient

**DOI:** 10.3389/fmed.2025.1677156

**Published:** 2025-11-17

**Authors:** Qiuping Zhang, Qiangjin Gong, Xiaokui Sun, Huizhi Zhu

**Affiliations:** 1 The First Clinical Medical College of Anhui University of Chinese Medicine, Hefei, China; 2 Shuguang Anhui Hospital Affiliated to Shanghai University of Traditional Chinese Medicine, Hefei, China; 3 The First Affiliated Hospital of Anhui University of Chinese Medicine, Hefei, China

**Keywords:** pulmonary nocardiosis, *Nocardia cyriacigeorgica*, infection, immunocompetent patient, Hemoptysis

## Abstract

Pulmonary nocardiosis is frequently missed or misdiagnosed due to its atypical clinical symptoms and non-specific imaging findings. Moreover, delayed diagnosis and treatment can lead to high mortality rates, underscoring the need to enhance etiological diagnosis. Here, we report a 55-year-old immunocompetent woman who developed pulmonary *Nocardia cyriacigeorgica* infection with massive hemoptysis as the initial symptom. The patient had no history of chronic respiratory diseases. Metagenomic next-generation sequencing of bronchoalveolar lavage fluid collected via bronchoscopy was performed, which confirmed the diagnosis. After targeted therapy with oral sulfamethoxazole-trimethoprim and linezolid, the patient achieved significant symptomatic and radiological improvement, accompanied by normalization of white blood cell count and neutrophil count. No recurrence was observed during follow-up.

## 1 Introduction

Nocardiosis is a suppurative granulomatous disease caused by Nocardia species infection. The condition affects multiple organs including the lungs, skin, and central nervous system, especially among immunocompromised patients (1, 2). Pulmonary nocardiosis (PN) is the most common infection (3, 4), primarily caused by inhalation of environmental Nocardia spores, with rare human-to-human transmission (5). In recent years, advancements in laboratory diagnostic techniques and identification methods, coupled with the rising immunodeficient population, have caused a significant increase in the number of reported cases (6). Nevertheless, its non-specific clinical manifestations and atypical imaging features, combined with the prolonged turnaround time of traditional culture methods, lead to delayed or missed diagnosis (7).

Here, we report a rare case of PN presenting with massive hemoptysis as the initial symptom. The patient was a 55-year-old immunocompetent woman without identifiable risk factors. The pathogen, identified through bronchoalveolar lavage fluid (BALF) culture and metagenomic next-generation sequencing (mNGS), was confirmed as *Nocardia cyriacigeorgica*. This species was first reported in 2001 (8), and generally infects immunocompromised individuals or patients with chronic lung diseases (9), and it is rare in immunocompetent hosts. The patient underwent bronchial artery embolization for massive hemoptysis, which resolved the bleeding but the progressive pulmonary lesions persisted. The infection was finally treated through a combination of interventional therapy, antibiotics, and intrapleural medication.

## 2 Case presentation

**Figure 1:**
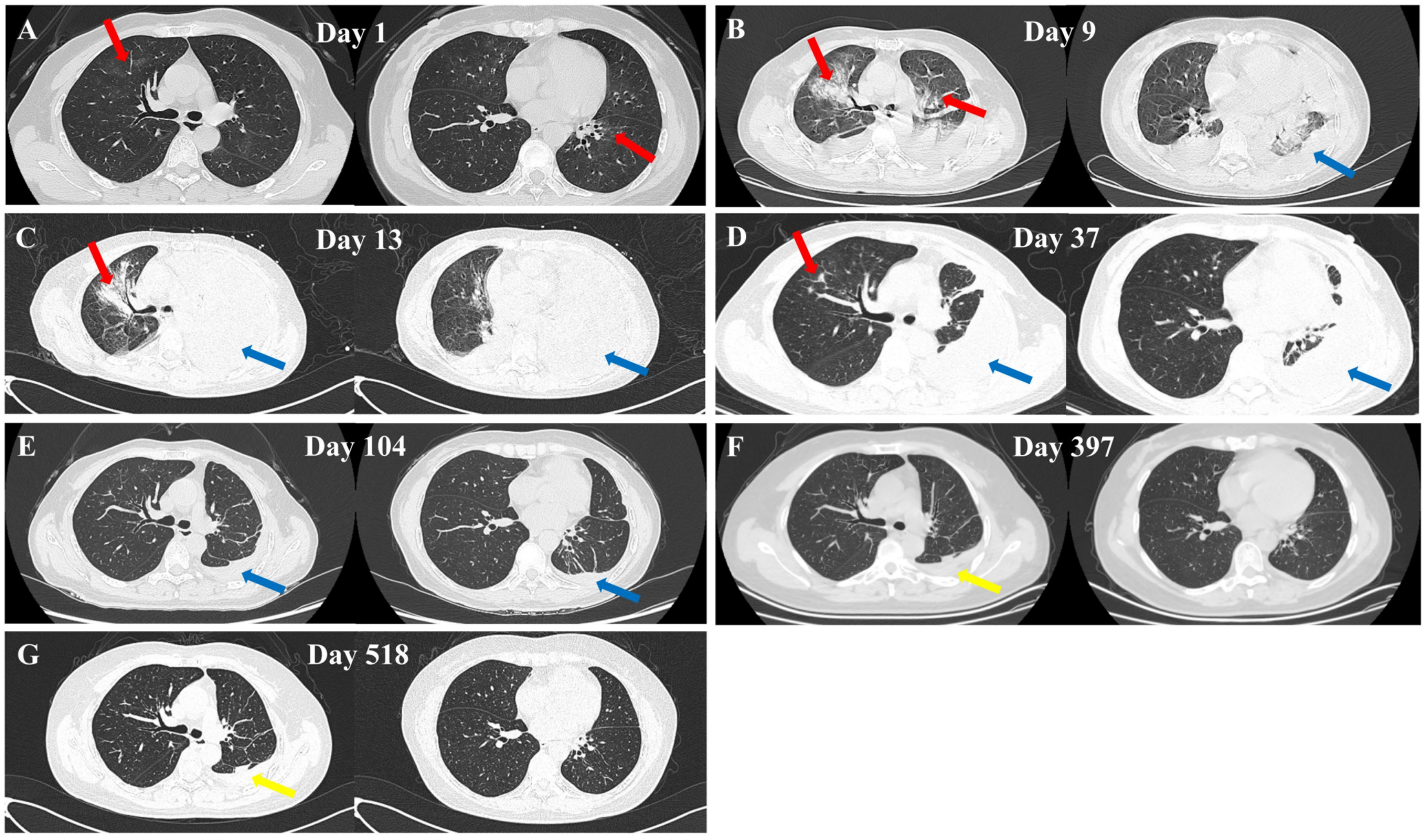
Chest CT images of the patient at different dates. **(A)** February 7, 2024 – scattered bilateral pulmonary infections (red arrows). **(B)** February 15, 2024 – multiple bilateral inflammatory lesions (red arrows) and pleural effusion (blue arrows). **(C)** February 19, 2024 – right lung inflammation (red arrow) and left lung pleural effusion with atelectasis (blue arrows). **(D)** March 15, 2024 – mild right lung inflammation (red arrow) and left lung pleural effusion with atelectasis (blue arrows). **(E)** May 21, 2024 – small amount of left-sided pleural effusion (blue arrows). **(F)** March 11, 2025 – left pleural thickening (yellow arrow). **(G)** July 9, 2025 – left pleural thickening (yellow arrow).

**Figure 2:**
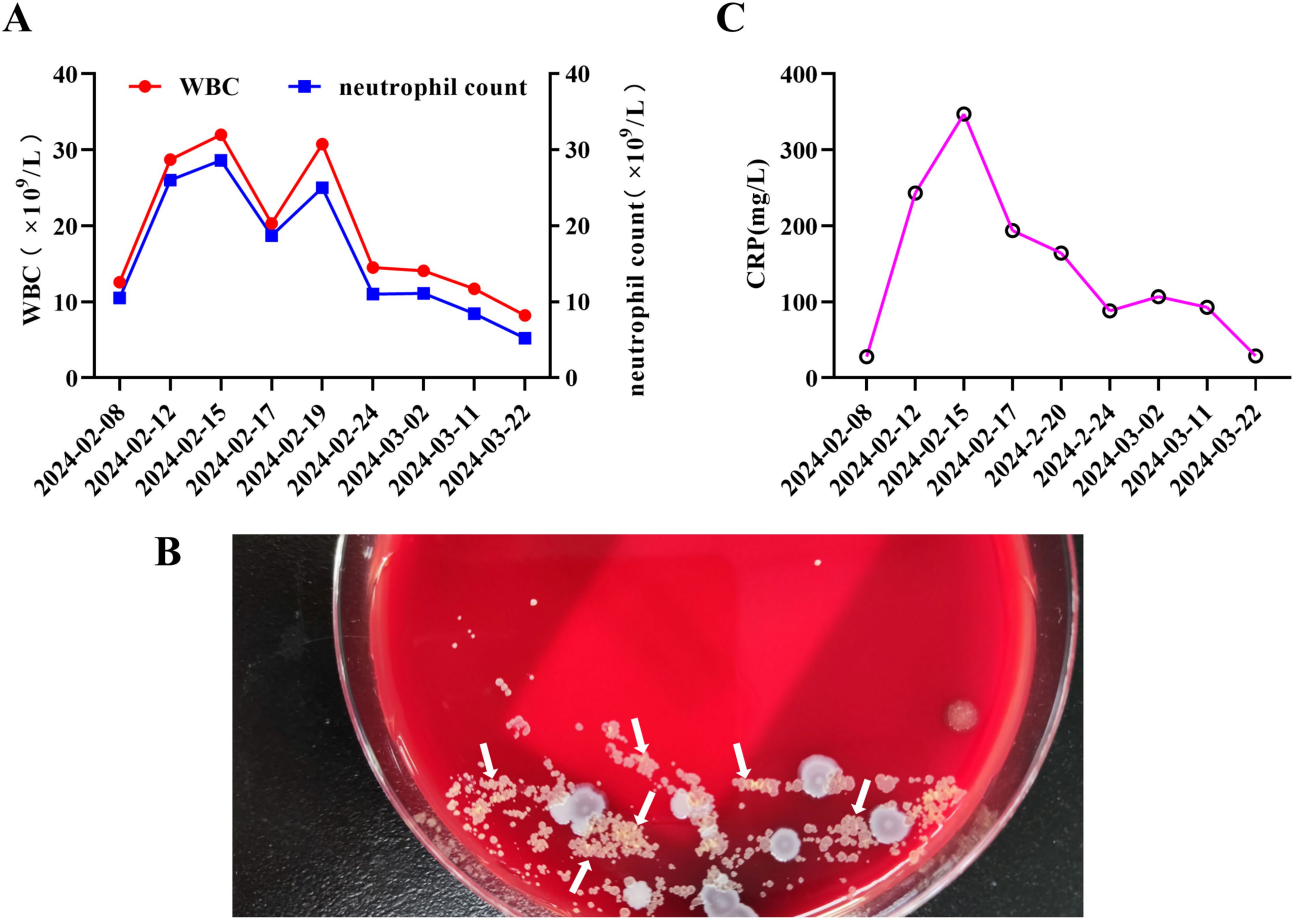
Clinical laboratory indicators and microbiological dvidence. **(A)**Trend curve of WBC, and neutrophil count during patient hospitalization. **(B)** Colonial morphology of *Nocardia* spp. on blood agar plate after 5-day culture of BALF (white arrows). **(C)** Trend curve of CRP during patient hospitalization.

A 55-year-old female farmer was admitted to a local hospital on February 7, 2024, due to sudden massive hemoptysis 12 h. She ceased farming over a decade ago and has not engaged in any work involving contact with soil, humus, or organic waste in recent times. She denied recent travel history, keeping pets, or visiting epidemic areas. She reported no history of smoking or alcohol consumption and no record of immunosuppressant use. Additionally, she had no significant past medical history of chronic obstructive pulmonary disease, asthma, hypertension, diabetes, or other major conditions. Chest CT on the day of presentation revealed localized bronchial dilation in the left lower lobe with scattered infections in both lungs (Figure 1A). On February 8, 2024, the complete blood count showed a white blood cell count (WBC) of 12.57 × 10^9^/L (reference range: 3.5–9.5 × 10^9^/L), neutrophil count of 10.5 × 10^9^/L (reference range: 1.8–6.3 × 10^9^/L), neutrophil percentage of 83.60%(reference range: 40–75%), red blood cell count (RBC) of 4.08 × 10^12^/L(reference range: 3.8–5.1 × 10^12^/L) (Table 1; Figure 2A). Preliminary diagnosis: bronchiectasis with hemoptysis. The patient received conservative management but it did not resolve the symptoms, and thus, bronchial artery embolization was conducted on February 11, 2024. Subsequently, post-procedure hemoptysis resolved, however, the patient still presented with worsening symptoms of cough, chest pain, and dyspnea, accompanied by low-grade fever. A repeat CT was performed on February 15, 2024 uncovered marked disease progression characterized by bilateral patchy consolidations and pleural effusions (Figure 1B). By February 19, 2024, the patient presented with altered mental status and was transferred to the ICU of The First Affiliated Hospital of Anhui University of Chinese Medicine.

**Table 1:** Patient’s blood routine test results.

Laboratory tests	Reference range	Results (Different test times)								
		Feb 8	Feb12	Feb15	Feb17	Feb19	Feb24	Mar 2	Mar11	Mar22
WBC(×10^9^/L)	3.5–9.5	12.57	28.7	31.98	20.33	30.76	14.53	14.07	11.71	8.23
RBC(×10^12^/L)	3.8–5.1	4.08	3.22	3.34	3.17	3.52	3.28	3.03	2.83	2.84
Hb(g/L)	115–150	119	93	94	91	103	94	87	82	81
Neutrophil(×10^9^/L)	1.8–6.3	10.5	26.0	28.6	18.7	25.01	11.01	11.13	8.46	5.22
Lymphocyte(×10^9^/L)	1.1–3.2	1.9	1.3	1.7	1.2	4.66	2.34	1.89	2.40	2.40
Eosinophil(×10^9^/L)	0.02–0.52	0.01	0.00	0.00	0.00	0.00	0.03	0.02	0.04	0.02
Basophil(×10^9^/L)	0–0.06	0.01	0.00	0.00	0.00	0.08	0.02	0.02	0.02	0.02
Monocyte(×10^9^/L)	0.1–0.6	0.16	1.46	1.69	0.45	1.01	1.04	1.01	0.79	0.57

On February 19, 2024, during the patient’s visit to our hospital, the physical examination revealed an axillary temperature of 37.4 °C (normal range: 36–37 °C), blood pressure of 120/75 mmHg (normal: <120/80 mmHg), pulse rate of 93 beats per minute (normal: 60–100 bpm), respiratory rate of 33 breaths per minute (normal: 12–20 bpm), and peripheral blood oxygen saturation of 90% (normal: ≥95%). Auscultation detected diminished breath sounds over the left lung and scattered dry/wet rales over the right lung. Emergency blood tests revealed the following results: WBC of 30.76 × 10^9^/L, neutrophil count of 25.01 × 10^9^/L, neutrophil percentage of 81.30%, and RBC of 3.52 × 10^12^/L. Pulmonary Artery Computed Tomography Angiography did not show any pronounced abnormalities. Results of the Chest CT demonstrated patchy high-density opacities in the right lung and a large left pleural effusion (Figure 1C). On the same day, a left-sided thoracentesis and drainage procedure was performed to collect pleural fluid samples for further analysis (Table 2). Given the rapid progression of the condition, a broad-spectrum antibiotic regimen consisting of meropenem and moxifloxacin was administered. Considering the potential presence of a specific pathogen infection, bronchoscopy was performed on February 21, 2024, to collect a BALF sample for mNGS analysis. On February 22, 2024, the patient showed signs of improvement, including regaining consciousness, fever subsiding, decreased respiratory rate, and relief of respiratory distress symptoms. Consequently, she was transferred from the intensive care unit to the respiratory medicine ward. Concurrently, the February 22, 2024, mNGS detected *Nocardia cyriacigeorgica* in BALF (Table 3), which was further confirmed through bacterial culture (Figure 2B). Other tests such as blood cultures, TB assays, viral/fungal panels, pleural fluid culture were negative.

**Table 2:** Pleural fluid analysis.

Parameters	Results	Reference range (Clinical significance)	Test result
Color	Turbid, Yellow	-	-
Rivalta test	Positive	-	Positive
WBC	2,560 × 10^6^/L	<100 × 10^6^/L(transudate); >500 × 10^6^/L (exudate)	Elevated
Total protein	38.6 g/L	<30 g/L(transudate); >30 g/L (exudate/infection)	Elevated
Lactate dehydrogenase	1,414 U/L	>500 U/L (malignant/infection)	Elevated
Glucose	1.56 mmol/L	<3.3 mmol/L (infection/tuberculous)	Decreased
Adenosine deaminase	11.6 U/L	>45 U/L(tuberculous)	Decreased

**Table 3:** Result of mNGS in BALF.

Gram’s staining	Genus		Species			
	Genus name	Sequences	Species name	Confidence level	Sequences	Relative abundance
G+	Nocardia	71	*Nocardia cyriacigeorgica*	High	71	79.05%

**Figure 3:**
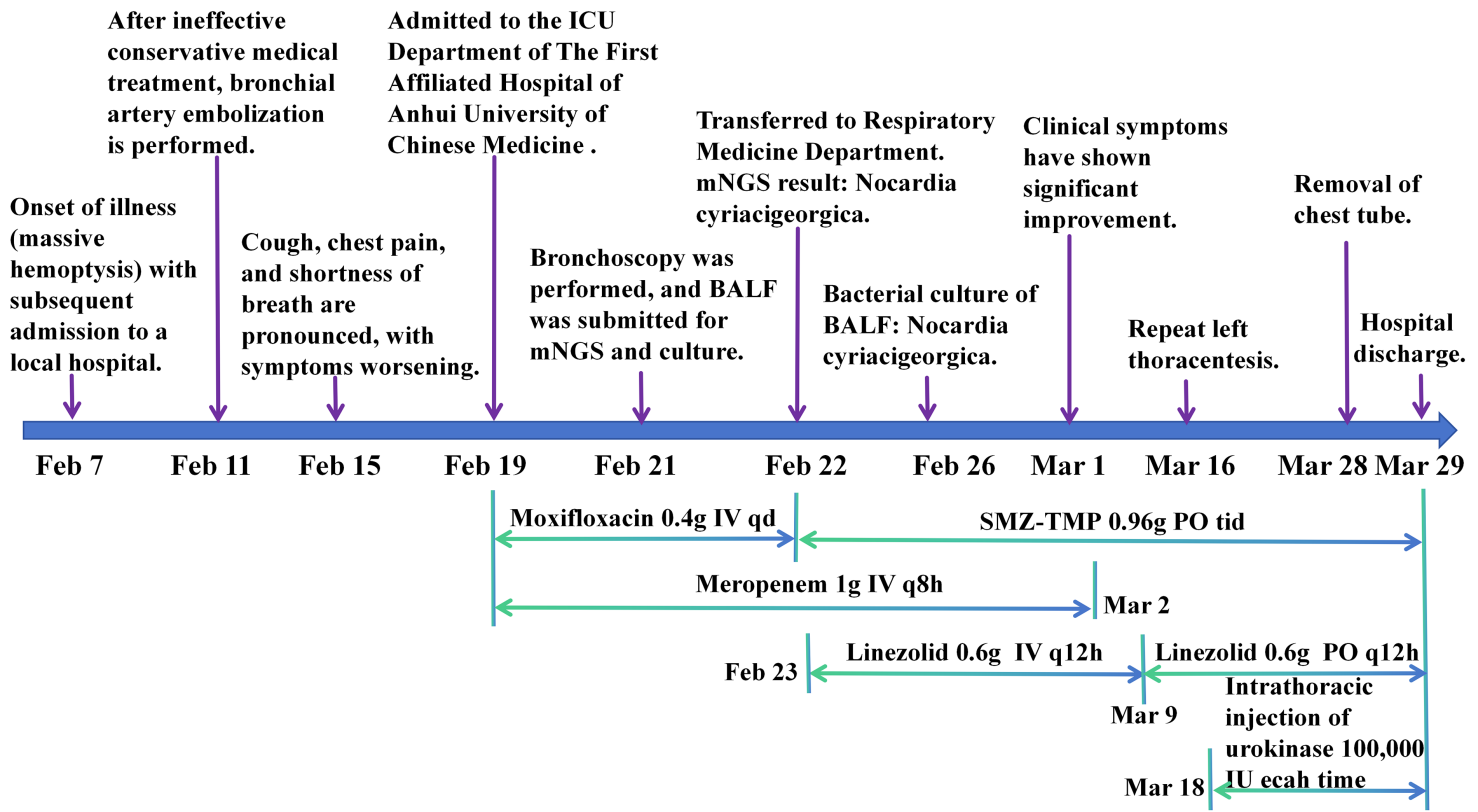
Treatment flow diagram.

Based on the patient’s clinical presentation, imaging findings, and mNGS results, a definitive diagnosis of pulmonary Nocardia infection was established. Starting February 22, 2024, the patient’s antimicrobial regimen was adjusted to: intravenous meropenem 1 g q8h for 12 consecutive days; oral sulfamethoxazole-trimethoprim (SMZ-TMP) 0.96 g tid for a total treatment duration of 6 months; Linezolid was initially administered intravenously at 0.6 g q12h for 15 days, followed by oral administration at 0.6 g q12h until the completion of the overall antimicrobial therapy regimen (total duration: 6 months). During the patient’s hospitalization, repeated monitoring of WBC, neutrophil count, and CRP showed gradual decreases in values with treatment (Figures 2A,C). On March 15, 2024, a follow-up chest CT revealed significant resolution of right lung lesions and a left-sided encapsulated pleural effusion (Figure 1D). To promote fluid absorption, a repeat thoracentesis was performed on March 16, 2024, with intermittent intrapleural administration of urokinase (100,000 IU per session, totaling 10 sessions). The patient was discharged on March 29, 2024, with recommendations for regular follow-up examinations. The entire treatment course was illustrated in Figure 3.

During follow-up, a chest CT scan on May 21, 2024 showed complete resolution of the right-sided lesion, with only minimal residual pleural effusion on the left side (Figure 1E). A follow-up chest CT on March 11, 2025 demonstrated complete resolution of the pleural effusion and left-sided pleural thickening (Figure 1F). A follow-up chest CT on July 9, 2025, showed no significant changes compared to the previous scan (Figure 1G). The patient reported no symptoms such as coughing or sputum production. Clinically, the patient has met the criteria for cure.

## 3 Discussion

PN is a suppurative or granulomatous lung disease caused by infections by the Nocardia species. It is considered a rare opportunistic infection, predominantly affecting immunocompromised individuals or those with chronic underlying conditions (10). So far, 119 Nocardia species have been identified, among which over 40 are pathogenic to humans (11). The most clinically prevalent species include: *Nocardia nova*, *Nocardia farcinica*, *Nocardia cyriacigeorgica*, *Nocardia brasiliensis*, and *Nocardia abscessus*. In the present case, the pathogen was identified as *Nocardia cyriacigeorgica*, an uncommon opportunistic bacterium in which is often detected in immunosuppressed hosts (12). This case was a 55-year-old female patient with no history of smoking, alcohol consumption, or immunosuppressive therapy. Her initial presentation was sudden massive hemoptysis, suggesting that Nocardia can present with an acute, progressive course even in hosts with relatively intact immune function, making this case report of significant value.

Most patients with PN show non-specific symptoms such as fever, cough, dyspnea, chest pain, and weight loss (7). The present case featured massive hemoptysis as the initial presentation, which is uncommon. Following bronchial artery embolization, the patient’s hemoptysis improved, but subsequently developed common pulmonary symptoms including cough, dyspnea, and chest pain, which progressively worsened, indicating disease progression. Although PN usually follows a subacute/chronic course (13), the present immunocompetent patient demonstrated an acute progression, with massive hemoptysis onset despite no prior respiratory symptoms, which is an exceptionally rare presentation.

Repeated blood tests revealed markedly elevated WBC, neutrophil count, and CRP levels, indicating a strong inflammatory response consistent with literature reports (7, 14). However, these laboratory markers also lack specificity. In this case, Table 1 showed that an immunocompetent patient with pulmonary *Nocardia cyriacigeorgica* infection exhibited markedly elevated WBC, neutrophil count, and monocyte count. As antimicrobial therapy progressed, WBC, neutrophil, and monocyte counts gradually returned to normal ranges, indicating effective control of the infection. Lymphocyte fluctuations were minimal, potentially reflecting differences between immunocompetent and immunosuppressed patients. Eosinophil counts remained persistently below the lower normal limit throughout the course, suggesting this infection was not allergy- or parasite-related. However, PN chest imaging also presents with diverse manifestations, including patchy opacities, nodular shadows, consolidation, nodules, masses, cavities, pleural thickening, and pleural effusion (15). These findings lack specific changes. When combined with nonspecific clinical symptoms, they frequently lead to misdiagnosis or missed diagnosis, often being mistaken for pulmonary tuberculosis, pulmonary fungal infections, or tumors. Notably, the pleural effusion reported in this case was encapsulated pleural effusion, consistent with findings in some literature reports (16). Pleural fluid analysis revealed markedly elevated WBC, total protein, and lactate dehydrogenase, characteristic of an exudative infectious effusion. These findings suggest that *Nocardia cyriacigeorgica* exhibits strong pleural invasiveness, involving both the pleura and pleural cavity. After local intrapleural administration of the drug, the effusion was completely reabsorbed. This suggests that in clinical practice, patients with rapidly progressing pulmonary infection complicated by pleural effusion should undergo active pleural fluid aspiration for diagnostic testing.

Rapid and accurate pathogen localization is critical for treatment. The accurate diagnosis of PN is based on the isolation of Nocardia from the specimen, and traditional diagnosis is often based on histopathologic examination and/or culture. However, Nocardia exhibits a slow growth, and the formation of visible colonies by Nocardia generally takes 2–7 d, with some genera requiring even several weeks of culture (17), and routine culture usually produces false-negative results, which increases the risk of delayed diagnosis and delayed initiation of targeted therapy. mNGS is a highly sensitive, high-throughput assay that can rapidly and objectively detect all pathogenic microorganisms in clinical samples. Compared to traditional culture methods, this approach provides shorter detection times and improved accuracy (10, 18, 19). In the case reported here, the time from sample collection to report was only one day. This enabled the initiation of anti-infective therapy during the critical window period when the patient’s pulmonary symptoms were worsening, thereby preventing further destruction of lung tissue. Moreover, its diagnostic performance remains unaffected by prior antibiotic use (20, 21). Sputum remains the most commonly used respiratory specimen for Nocardia isolation. However, BALF, due to its minimally invasive procedure, ensures a low contamination rate in specimens, yielding more reliable diagnostic results—particularly in critically ill or immunocompromised patients with pulmonary infections (22). Studies have shown that the prognosis of PN is poor, with a case fatality rate estimated at approximately 41% (23). A Japanese study reported a case fatality rate of 56.7% (24), implying that early diagnosis is crucial to accelerate the timely application of treatments. In this case, the rapid targeting of the pathogen by BALF mNGS allowed early initiation of the targeted treatment, and the subsequent BALF culture was also verified, which significantly improved the patient’s prognosis.

Regarding treatment, no randomized controlled clinical trials have been performed to test treatments for nocardiosis. The selection of antimicrobial agents and treatment duration require comprehensive consideration of the infection site and severity, Nocardia species, host immune status, drug susceptibility testing, as well as tolerance and adverse reactions to antibiotics (2). Early diagnosis and prompt initiation of appropriate therapy are advocated to improve PN curation rates and reduce mortality. Sulfonamide drugs represent the first-line treatment for nocardiosis, with trimethoprim-sulfamethoxazole (TMP-SMX) being the most thoroughly studied and preferred therapeutic agent (25, 26). Antibacterial agents such as imipenem, amikacin, and linezolid are also recommended for the treatment of nocardiosis (2). Linezolid has been reported to exhibit high sensitivity against all Nocardia species with significant therapeutic efficacy (27, 28). For severe or disseminated disease, combination therapy with at least two antimicrobial agents is recommended. Pulmonary infections require at least 6 months of treatment. However, the exact duration is customized to the type of selected regimen and the patient’s therapeutic response (2). Antibiotic susceptibility varies among different *Nocardia* spp. For example, *Nocardia cyriacigeorgica* is sensitive to TMP-SMX, moxifloxacin, imipenem, linezolid, amikacin, and tobramycin, but resistant to ciprofloxacin, clarithromycin, and amoxicillin-clavulanic acid (2). Initial treatment for this case involved meropenem, SMZ-TMP combined with linezolid for Nocardia infection. Treatment was subsequently adjusted to a combination of SMZ-TMP and linezolid for a total duration of 6 months. Follow-up chest CT scans during treatment demonstrated progressive resolution of the lesions. Regular follow-up was conducted, and post-treatment imaging revealed complete resolution of the lesions. Clinical and radiographic evaluations confirmed no recurrence of the disease, ultimately achieving clinical cure.

## 4 Summary

Our case report demonstrates that acute severe pulmonary nocardiosis can occur in immunocompetent individuals, and presents with massive hemoptysis as the initial manifestation. The integration of mNGS with conventional diagnostic techniques is recommend to achieve early and precise diagnosis, and treatment approaches involving a combination of interventional hemostasis, targeted antibiotic therapy, and local drainage is ideal for such patients. In clinical practice, for patients with rapidly progressive pneumonia and pleural effusion, even if they present with normal immunity, the possibility of pulmonary Nocardia infection should be considered, and mNGS testing should be performed as early as possible. In patients with lower respiratory tract infections who exhibit pulmonary involvement, poor response to standard antimicrobial therapy, atypical imaging features, and rapid disease progression, pulmonary Nocardia infection should be considered. Early implementation of mNGS is recommended to support timely diagnosis and guide appropriate treatment.

## Data Availability

The original contributions presented in the study are included in the article/supplementary material, further inquiries can be directed to the corresponding author/s.
